# Co-inoculation of fungi and desert cyanobacteria facilitates biological soil crust formation and soil fertility

**DOI:** 10.3389/fmicb.2024.1377732

**Published:** 2024-04-08

**Authors:** Xiangjun Zhou, Bin Liang, Tian Zhang, Qiao Xiong, Xiao Ma, Lanzhou Chen

**Affiliations:** ^1^Huangshi Key Laboratory of Prevention and Control of Soil Pollution, College of Urban and Environmental Sciences, Hubei Normal University, Huangshi, China; ^2^Hubei Key Laboratory of Biomass-Resources Chemistry and Environmental Biotechnology, School of Resource and Environmental Sciences, Wuhan University, Wuhan, China

**Keywords:** biological soil crusts, co-inoculation, fungi, cyanobacteria, dissolved organic matter

## Abstract

The inoculation of cyanobacteria for enriching soil nutrients and forming biological soil crusts (BSCs) is considered an effective means to restore degraded soil. However, there are limited studies on the application of co-inoculation of fungi and cyanobacteria for degraded soil remediation. In this study, a high exopolysaccharide-secreting fungi Zh2 was isolated from lichen BSCs in Hobq Desert, and co-inoculated with a cyanobacterial strain identified as *Phormidium tenue* in different proportions to form BSCs on sand during a 35 days incubation period. Results revealed significant differences in crust biomass and soil properties among crusts with different cyanobacterial/fungal inoculation ratios. Microbial biomass, soil nutrient content and enzyme activities in crusts co-inoculated with cyanobacteria and fungi were higher than those inoculated with cyanobacteria and fungi alone. The inoculation of cyanobacteria contributed to the fulvic-like accumulation, and the inoculated fungi significantly increased the humic-like content and soil humification. Redundancy analysis showed that the inoculation of cyanobacteria was positively correlated with the activities of urease and phosphatase, and the content of fulvic-like. Meanwhile, the inoculation of fungi was positively correlated with the contents of total carbon, total nitrogen and humic-like, the activities of catalase and sucrase. Cyanobacteria and fungi play distinct roles in improving soil fertility and accumulating dissolved organic matter. This study provides new insights into the effects of cyanobacteria and fungi inoculations on the formation and development of cyanobacterial-fungus complex crusts, offering a novel method for accelerating induced crust formation on the surface of sand.

## 1 Introduction

Desertification is a serious global environmental challenge, with approximately 40% of the Earth’s land experiencing its adverse effects, imperiling the livelihoods, and living conditions of a billion individuals (Dregne et al., 1991; Verón et al., 2006). This phenomenon is exacerbated by climate change, overgrazing, unsustainable agricultural practices, and the excessive exploitation of water resources (Wang et al., 2023; Ren et al., 2024). In China, the government has adopted the “Grain for Green” program (Segura et al., 2020) and “Soil and Water Conservation Engineering” (Karlen et al., 2014; Li et al., 2020). These initiatives involve measures such as reducing agricultural activities, slope protection, afforestation, artificial grass planting, and windbreak construction. These engineering projects entail substantial economic investment and manual maintenance (He et al., 2023), yet the desertification recovery process remains slow. The development of desertification control technology continues to be a global concern.

Biological soil crusts (BSCs) living within or on top of the uppermost millimeter of soil are soil particle-associated communities of cyanobacteria, algae, fungi, lichens, liverworts, mosses and bacteria in different proportions, and form coherent layers at the soil surface (Belnap et al., 2016; Ferrenberg et al., 2017). BSCs have long been known as “pioneers” or “ecosystem engineers” in drylands because of their multifunction on water reservation, carbon and nitrogen cycles, soil stabilization and preparation for the establishment and performance of vascular plants (Bowker et al., 2014; Rodriguez-Caballero et al., 2022; Marasco et al., 2023). BSCs are widely distributed in semiarid and arid areas, which constitute up to 40% of the terrestrial land surface (Weber et al., 2016). As 10–20% of these drylands are currently suffering from severe degradation (Maestre et al., 2016), the restoration of drylands is an urgent issue and one way to achieve this goal can be through the rehabilitation of BSC (Bowker, 2007; Antoninka et al., 2020; Rossi et al., 2022).

The biological crust can be classified into algae crust, lichen crust, and moss crust based on the primary components present in the biological soil crust (Weber et al., 2022). Among them, the lichen crust is a typical symbiont formed by cyanobacteria or green algae and fungi, performing multiple functions derived from a variety of microbial attributes and metabolic pathways (Li et al., 2017; Grimm et al., 2021). Cyanobacteria or green algae in lichen contribute energy and nutrients to the symbiotic organism through photosynthesis and nitrogen fixation, while fungi utilize mycorrhizae to absorb water and inorganic salts from cyanobacteria or green algae. Additionally, fungi play a role in converting inorganic substances into organic matter through saprophytic decomposition (Cao et al., 2024). The fungal hyphae can envelop algal cells, providing protection against external stress, and their tightly interwoven structure facilitates the storage of air and moisture. Lichen crusts have been reported to be more efficient to algal crusts in terms of soil improvement, fertilization, windbreak, and sand fixation (Atashpaz et al., 2023).

Previous studies have demonstrated that cyanobacterial inoculation could assist in induced BSC (IBSC) formation and development by overcoming crust organisms’ dispersal limitation (Lan et al., 2014; Chamizo et al., 2018; Kheirfam et al., 2020). There are numerous works demonstrating successful cultivation of mosses (Antoninka et al., 2016; Bu et al., 2018; Liao et al., 2024), but very limited works on lichen cultivation. One study involved the translocation of lichens from a quarry to a degraded gypsum area, where researchers tested various adhesives to improve lichen attachment to the substrate, to enhance the restoration of gypsum soils (Ballesteros et al., 2017). Bacterial and fungal inoculants can be considered as potential choices in strategies for restoring degraded soils (Rashid et al., 2016). The addition of nitrogen-fixing bacteria (Rezasoltani and Champagne, 2023), phosphorus-solubilizing bacteria (Liu et al., 2023), mycorrhizal fungi (Chaudhary et al., 2020) and fungi with high extracellular polysaccharide production significantly enhances soil fertility (Rashid et al., 2016; Mao et al., 2024). Notably, the incorporation of fungi with high extracellular polysaccharide production not only increases soil organic matter but also forms aggregates by secreting extracellular polymers, effectively binding soil particles and minerals. The co-inoculation of cyanobacteria and high extracellular polysaccharide-producing fungi holds the potential to enhance the conversion of soil organic matter and facilitate the restoration of degraded soils in arid regions.

The structural composition and stability of soil organic matter are important indicators of desertification soil restoration (Jensen et al., 2020). Soil microorganisms could convert new carbon in the soil into stable carbon, i.e., humus, through their metabolic activities, thereby improving soil ecological functions (Yang et al., 2022). Dissolved organic matter (DOM) in the soil mainly originates from plant residues, root exudates, and microbial metabolic activities and is the most active component of soil organic matter. DOM consists of highly heterogeneous components with different molecular weights and structures, which not only affect the bioavailability of soil nutrients such as carbon, nitrogen, sulfur, and phosphorus but also play a significant role in microbial growth, metabolism, and soil formation processes (Li et al., 2023). Liu et al. (2021) used three-dimensional fluorescence spectroscopy to investigate soil DOM biodegradation at different successional stages of vegetation recovery, demonstrating the sensitivity of DOM as an indicator reflecting the degree of soil recovery. Swenson et al. (2015, 2018) revealed the link between microbial metabolism and soil environmental chemistry by understanding the DOM composition and abundance changes in the inoculation experiment. Assuming that the inoculated microorganisms contribute to desert soil nutrition, DOM composition and characteristics can respond quickly and potentially reveal differences in the roles of cyanobacteria and fungi.

This study draws inspiration from the lichen mutualistic symbiosis model to investigate the effects of different proportions of filamentous cyanobacteria and high-yield exopolysaccharide fungi inoculation on: (i) the growth of IBSC and variations in cyanobacterial and fungal biomass; (ii) improvements in soil properties, including carbon, nitrogen, and soil enzyme activity; and (iii) alterations in soil dissolved organic matter (DOM) composition and characteristics. The objective is to elucidate the contributions of cyanobacteria and fungi to the soil restoration process and to provide a novel method for accelerating IBSC formation on the surface of sand.

## 2 Materials and methods

### 2.1 Soil collection

The soil samples were obtained from moving sandy dunes in Hobq Desert, Dalateqi County, Inner Mongolia, China (44°21′N; 109°50′E). The Hobq Desert is a hyper plateau at an elevation of 1,230 m and characterized by mass moving sandy dunes in an arid to semi-arid climate (Chen et al., 2014). The average annual temperature is 7.4°C, and the prevailing wind direction is WNW, with an average annual speed of 2.7 m/s. The mean annual evaporation (2,448 mm) far exceeds the mean annual precipitation (293 mm). Sand samples was carried from 10 cm of the surface layer. The sand was air-dried and sieved by 2 mm-sized mesh. Subsequently, 500 g sand were analysed for soil physicochemical properties (Supplementary Table S1), and the remaining portion was utilized as soil substrates for inoculation. Additionally, there are experimental areas where cyanobacteria were inoculated onto the sandy surface between 2002 and 2008 for desertification control, as documented in our previous study (Deng et al., 2020). Dark lichen BSCs were gathered from this experimental area to screen strains.

### 2.2 Screening and taxonomic identification of strain

Dark lichen BSCs with three replicates were added to flasks with enrichment medium (5 g/L tryptone, 5 g/L yeast extract, 1 g/L K_2_HPO_4_, and 1 g/L glucose), and shaken at 30°C for 20 min to obtain the soil suspension. Next, the microbial suspension was diluted with sterile water at a ratio of 1: 10, and further diluted to 10^−6^. Afterwards, 200 μL of the solutions from dilution gradients of 10^−4^, 10^−5^, and 10^−6^ were evenly spread onto PDA, Ashby nitrogen-free, and phosphate-solubilizing solid media. The plates were then inverted and incubated at 30°C in a constant temperature incubator for 24 h. Following incubation, distinct single colonies of various types were selected, streaked multiple times for purification on agar plates, and preserved at 4°C on PDA slants for future use.

To test the ability of the strain to produce exopolysaccharides, 2 volumes of 95% ethanol was added to the fermentation, and after 2 h reaction, the mixture was centrifuged at 7000 rpm for 10 min. Next, 1 mL deionized water was added to dissolve the precipitate and the solution was fully reacted in a boiled water bath for 5 min after added 1 mL DNS reagent. The strain in which the cooled solution turned reddish brown was recognized as a polysaccharide producing strain. One highly polysaccharide-excreting fungus designated Zh2 was isolated and used for further research.

The fungal Zh2 was identified by sequencing 18S rDNA gene. DNA extraction was carried out following the instruction of Genomic DNA Extraction Kit (TianGen, China). The universal primers used in PCR amplification were as follows: ITS1 (5’-TCCGTAGGTGAACCTGCGC-3′) and ITS4 (5’-TCCTCCGCTTATTGATATGC-3′). The amplication results were sequenced by the BGI Gene Co. (Wuhan, China). A BLAST search was conducted at the National Center for Biotechnology Information (NCBI, https://www.ncbi.nlm.nih.gov/). Sequencing data were phylogenetically analysed using Mega 5 and submitted to NCBI database with the sequence number MH973233. The results indicated the strain Zh2 belongs to *Exophiala oligosperma* (Supplementary Figure S1).

The genus *Phormidium tenue*, affiliated with the order Oscilatoriaceae, is commonly found in BSC communities within arid regions (Hu et al., 2003). The strains were kindly provided by Institute of Hydrobiology, Chinese Academy of Sciences. It was isolated from the Tengger Desert in Shapotou, Zhongwei County, Ningxia, China (37°27′N, 104°57′E), and deposited at the Freshwater Algae Culture Collection at the Institute of Hydrobiology (FACHB-collection), with deposit number FACHB886. The genus *Phormidium tenue* has been successfully used for inoculation in the Hobq Desert (Xie et al., 2007). Additionally, it has been found to dominate in natural biocrusts in the Hobq Desert (Deng et al., 2020) and the Loess Plateau (Zhang et al., 2018). Furthermore, *Phormidium tenue* has been reported to perform well in resisting UV damage (Wang et al., 2017), water stress (Chen et al., 2012), and promoting shrub growth (Xu et al., 2013). Therefore, it is suitable for inoculation onto the surface of shifting sand dunes.

### 2.3 Inoculum preparation and cultivation

The *Exophiala oligosperma* Zh2 were inoculated into sterile PDA medium and cultured in a constant temperature shaking incubator at 28°C and 150 rpm for 48 h. After homogenizing, the filamentous cyanobacterium *Phormidium tenue* was transferred to sterile BG11 medium and cultured in a light incubator at a constant temperature of 25 ± 2°C under 24-h continuous illumination with a light intensity of 50 μEm^−2^ s^−1^ for 4 days with aeration. The fungal and cyanobacterial cultures were then centrifuged, the supernatant was discarded, and the pellets were washed several times with sterile water. The pellets were then resuspended in sterile water to serve as stock solutions for both cyanobacteria and fungi, and the concentrations were determined for subsequent inoculation.

For fungal biomass determination, the hemocytometer counting method was employed. Specifically, a small volume of well-shaken fungal suspension was drawn up with a pipette and dropped into the grooves on both sides of the central platform of a counting chamber. Excess fungal suspension in the grooves was removed using absorbent paper. After a brief settling period, the cells were counted under a microscope, and the results were expressed as CFU/mL.

The biomass of *Phormidium tenue* was characterized by the content of chlorophyll *a* (Chl *a*). The measurement method for Chl *a* in cyanobacteria was as follows: 5 mL of cyanobacterial solution was centrifuged at 8000 rpm for 10 min, the supernatant was discarded, and the algal cells were collected. Then, 5 mL of 95% ethanol was added, and the mixture was extracted and soaked in a refrigerator at 4°C for 24 h, shaking 2–3 times in between to ensure full dissolution of Chl *a*. The supernatant was collected after centrifugation (8,000 rpm, 10 min), and the absorbance values (OD) at 665 nm and 649 nm were measured using a UV-2000 spectrophotometer. The Chl *a* content was calculated using the formula: Chl *a* (mg/L) = 13.95 × OD_665_-6.88 × OD_649_.

Finally, based on the concentrations of the cyanobacterial and fungal stock solutions, the dilution factors were calculated, and the stock solutions were diluted to the desired concentrations using sterile water.

After the *Exophiala oligosperma* Zh2 and *Phormidium tenue* FACHB886 were cultured to the logarithmic phase, the biomass was determined by hemocytometer and Chl *a* method, respectively, as a stock solution. The stock solution was diluted to target concentration with sterile water and mixed to obtain a mixed cyanobacterial-fungal inoculum. Sand, sieved, sterilized, dried, and distributed into Petri dishes (90 mm × 12 mm), each containing 60 g, served as the growth substrate.

Five treatment groups were established, with 5 Petri dishes in each group as replicates. After extensive preliminary experiments, the inoculation amounts of cyanobacteria and fungi were set at 1 ± 0.1 μg (Chl *a*)/cm^2^ and 10^7^ CFU/cm^2^, respectively. Thus, the sand surface of the Cya group was inoculated with 1 ± 0.1 μg (Chl *a*)/cm^2^ of *Phormidium tenue*; the Cya + Fun group was simultaneously inoculated with 1 ± 0.1 μg (Chl *a*)/cm^2^ of *Phormidium tenue* and 10^7^ CFU/cm^2^ of the *Exophiala oligosperma* Zh2; The 2Cya + Fun group was simultaneously inoculated with 2 ± 0.15 μg (Chl *a*)/cm^2^ of *Phormidium tenue* and 10^7^ CFU/cm^2^ of the *Exophiala oligosperma* Zh2; The Cya + 2Fun group was simultaneously inoculated with 1 ± 0.1 μg (Chl *a*)/cm^2^ of *Phormidium tenue* and 2*10^7^ CFU/cm^2^ of the *Exophiala oligosperma* Zh2; the Fun group was inoculated with 10^7^ CFU/cm^2^ of the *Exophiala oligosperma* Zh2.

The inoculated Petri dishes were incubated in a light incubator at at a constant temperature of 25 ± 2°C under 24-h continuous illumination with a light intensity of 50 μEm^−2^ s^−1^. Throughout this period, water was added daily at 9:00 according to the soil moisture content to maintain a water content of 15%. The culture period spanned 35 days, during which samples were collected six times, weekly, to assess crust Chl *a* content, DNA content, cyanobacterial and fungal gene copy numbers, as well as soil properties and dissolved organic matter (DOM) characteristics.

### 2.4 Analysis of BSC biomass

Chl *a* extraction from 1.0 g of soil samples involved using 5 mL of ethanol at 80°C for 5 min. The samples were subsequently incubated at 4°C for 8 h and then centrifuged at 3500 × g for 15 min. The extraction process was repeated twice to ensure complete pigment recovery. The supernatant, containing the pigments, was measured at 655 nm and 750 nm using a spectrophotometer (UV-1700 PharmaSpec, Japan). The Chl *a* concentration was calculated using the formula reported by Mugnai et al. (2018).

Soil DNA was extracted from 0.5 g of soil of each BSC sample using a FastDNA Spin Kit for Soil (MP Biomedical, LLC) and an MP FastPrep-24 Instrument (MP Biomedical, LLC) according to the supplied protocols. DNA extracts were examined on 0.9% agarose gels in 1× Tris-acetic acid-EDTA with GelRed and quantified using a Nanodrop 2000c (Thermo Scientific, United States). The fungal 25-28S rRNA genes and cyanobacterial 16S rRNA gene were quantified with a StepOne Real-Time PCR System (Applied Biosystems, United States) using the primers NL1f and LS2r, and CYA359F and CYA781A/B (Deng et al., 2016), respectively. Further details of qPCR procedures were outlined in our previous study (Zhou et al., 2020).

### 2.5 Measurement of soil properties

Following the 35-day culture period, the crust was air-dried at 25°C. The cyanobacterial-fungal composite crust underwent analysis for its C/N/H element content, along with assessments of catalase, sucrase, urease, and phosphatase activities. Soil elemental content was determined using an elemental analyzer (Vario Macro Cube, Elementar, Germany). Soil enzyme activities were conducted according to the method described by references (Liu et al., 2014; Yang et al., 2018). Catalase activity in the soil was determined using the KMnO_4_ titration method, sucrase activity was assessed through the dinitrosalicylic acid colorimetric method, urease activity was measured via the sodium phenolate-sodium hypochlorite colorimetric method, and phosphatase activity was determined using the disodium benzene phosphate colorimetric method.

### 2.6 Fluorescence emission-excitation matrix (EEM) spectroscopy

Subsequently, 5.0 g of the sieved soil, mixed with 30 mL of deionized water, underwent shaking at 200 rpm for 30 min at 25°C. The resulting aqueous extracts were subjected to centrifugation and filtration to isolate the dissolved organic matter (DOM). The carbon content in DOM, also known as dissolved organic carbon (DOC), was measured using a TOC analyser (Vario TOC, Elementar, Germany) (Zhou et al., 2019). DOM fluorescence was measured in a 1.0 cm quartz cell using a spectrofluorometer (Edinburgh, FS5) equipped with continuous (150 W) and pulsed xenon lamps. Scanning ranges were configured at 200–500 nm with 5 nm sampling intervals for excitation (Ex), and at 250–600 nm with 5 nm sampling intervals in emission (Em). This setup allowed for comprehensive information gathering about all organic compounds present (Chen et al., 2003). Spectra were recorded at a scan rate of 1,200 nm/min with a dwell time of 0.01 s, utilizing excitation and emission slit bandwidths of 10 nm. The spectra underwent blank subtraction, correction for inner filter effects (for both excitation and emission wavelengths), and masking with first-and second-order Rayleigh scattering (±10 nm at ƛex = ƛem and 2ƛex = ƛem) (Fox et al., 2017). Deionized water blanks were analyzed under the same conditions as the samples.

In EEM spectra, three indices are commonly used to reflect the degree of humification and the bioavailability of DOM (Ye et al., 2020; Xue et al., 2022). The Humification Index (HIX) is used to characterize the content of humic substances. It is the ratio of the integrated values between the regions of Em 435–480 nm and Em 300–345 nm under an Ex of 254 nm. The Biological Index (BIX) refers to the ratio of fluorescence intensity at Em 380 nm and Em 430 nm when Ex is set to 310 nm, indicating the bioavailability of organic matter. The Fluorescence Index (FI) represents the ratio of fluorescence intensity at Em 470 nm and Em 520 nm when Ex is 520 nm, primarily reflecting the source of dissolved organic matter (DOM). Microbial activity is considered the main source of DOM when 1.7 < FI < 2.0, while microbial contribution is lower when FI is less than 1.5.

### 2.7 Statistical analyses

A one-way ANOVA was conducted to assess the significance of differences across the analyses for soil property indicators, including soil C/N content and enzyme activity. Tukey’s test (*p* < 0.05) was subsequently employed to ascertain the statistical significance of the variables (IBM SPSS Statistics v20). A repeated-measures ANOVA was used to determine the effects of cultivation time and treatment on Chl *a*, cyanobacterial and fungal abundance. Parallel Factor Analysis (PARAFAC) was conducted to separate distinct fluorescent components, and DOM indices, including FI, BIX, and HIX, were determined using MATLAB 7.0 (Mathworks, Natick, MA). The analysis utilized the DOM Fluor toolbox.^1^ The outcomes wwpere then compared with previous findings on soil environments accessible in the OpenFluor database.^2^ To investigate the effects of algae and fungi inoculation levels on the biomass of composite crusts and soil properties, the redundancy analysis (RDA) was conducted in R (Version 3.5.1, MathSoft, United States) with the “vegan” package. The inoculation levels of cyanobacteria and fungi were assigned values of 0, 1, 2, where the Cya group is denoted as Cya = 1, Fun = 0; the Cya + Fun group as Cya = 1, Fun = 1; the 2Cya + Fun group as Cya = 2, Fun = 1; the Cya + 2Fun group as Cya = 1, Fun = 2; and the Fun group as Cya = 0, Fun = 1. Data representation and graphical fittings were generated using OriginPro 2023.

## 3 Results

### 3.1 Growth of IBSCs with different inoculum biomass

**Figure 1:**
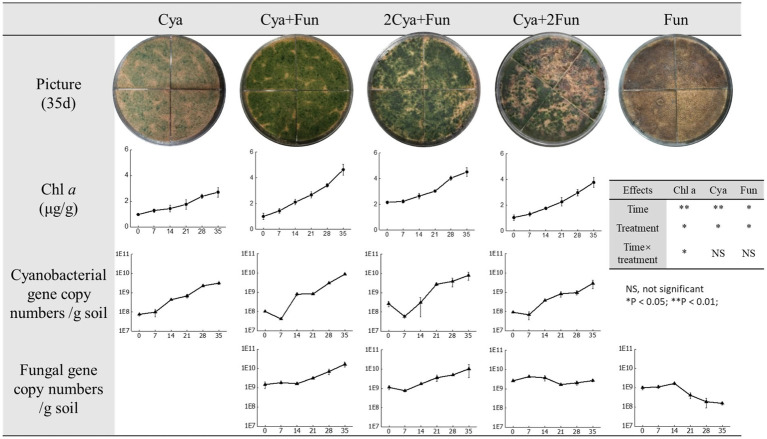
Images depicting crusts with varying inoculum biomass after 35 days of inoculation, along with the biomass growth curve during the culture period. Lines 4 and 5 represent changes in cyanobacterial and fungal gene copy numbers per gram of soil, respectively. The x-axis of the line chart corresponds to the cultivation time (days). Effects of cultivation time and treatment on Chl *a*, cyanobacterial and fungal abundance on the basis of the repeated-measures ANOVA analyses also shown.

As shown in Figure 1, both cyanobacteria and fungi exhibited viability in sand, forming IBSCs. Notably, the distribution of cyanobacterial coverage varied among crusts inoculated with different inoculum biomass. Cya and Cya + Fun were characterized by a more even layer, whereas Cya + 2Fun displayed a patchy distribution (Figure 1). A repeated-measures ANOVA showed that there were significant differences in Chl *a* content, cyanobacterial and fungal abundance between different cultural time or inoculation treatment. Cyanobacterial abundance in cyanobacteria-fungi co-inoculated crusts showed an adaptive decline after the initial inoculation. Throughout the 35-day cultivation period, the Chl *a* content and cyanobacterial abundance in crusts co-inoculated with cyanobacteria and fungi surpassed those in the singly inoculated cyanobacteria. Among all experimental groups, Cya + Fun exhibited the most rapid growth rate in terms of Chl *a* content and cyanobacterial abundance. The abundance of fungi in cyanobacteria-fungi co-inoculated crusts displayed an overall growth trend, with the growth rate slightly lower than that of cyanobacteria. While fungi could sustain growth in the co-inoculated scenario, the abundance of fungi was observed to gradually decrease in the later stages of cultivation when the fungus was inoculated alone.

### 3.2 Soil C, N and enzymes activity

After a 35-day inoculation period, significant differences in soil C, N content and enzyme activity were observed between crusts with varing cyanobacterial/fungal inoculation ratios (Table 1). The Cya + Fun group exhibited the highest soil C and N content, while the Cya group had the lowest. The treatments involving the combined inoculation of cyanobacterial and fungal strains showed a significant augmentation in both soil C, N content, and enzyme activity. Specifically, soil catalase, sucrase, urease, and phosphatase activities in crusts co-inoculated with cyanobacteria and fungi surpassed those of the Cya and Fun groups, which were singly inoculated with cyanobacteria or fungi. Notably, the Cya + Fun and Cya + 2Fun groups displayed elevated soil catalase and sucrase activities compared to other groups.

**Table 1:** Soil nutrient contents and enzyme activities in cyanobacteria-fungi crusts with varying inoculum biomass.

	Cya	Cya + Fun	2Cya + Fun	Cya + 2Fun	Fun
Soil C	1.01 ± 0.16c	1.33 ± 0.07a	1.12 ± 0.03bc	1.28 ± 0.04ab	0.97 ± 0.02c
Soil N	0.09 ± 0.01b	0.14 ± 0.02a	0.12 ± 0.02ab	0.12 ± 0.01ab	0.11 ± 0.01ab
Catalase activity (U/g)	5.27 ± 0.32c	6.21 ± 0.3ab	5.83 ± 0.06b	6.37 ± 0.11a	5.14 ± 0.07c
Sucrase activity (U/g)	0.34 ± 0.004e	0.47 ± 0.007b	0.42 ± 0.008c	0.48 ± 0.006a	0.38 ± 0.006d
Urease activity (U/g)	1.68 ± 0.05b	1.79 ± 0.04a	1.78 ± 0.02a	1.75 ± 0.05ab	1.39 ± 0.02c
Phosphatase activity (U/g)	1.39 ± 0.1c	1.59 ± 0.02b	1.93 ± 0.07a	1.5 ± 0.09bc	1.34 ± 0.07c

Different letters (a,b,c,d,e) indicate significant differences between treatment groups.

### 3.3 Soil DOM characteristics and components

**Figure 2:**
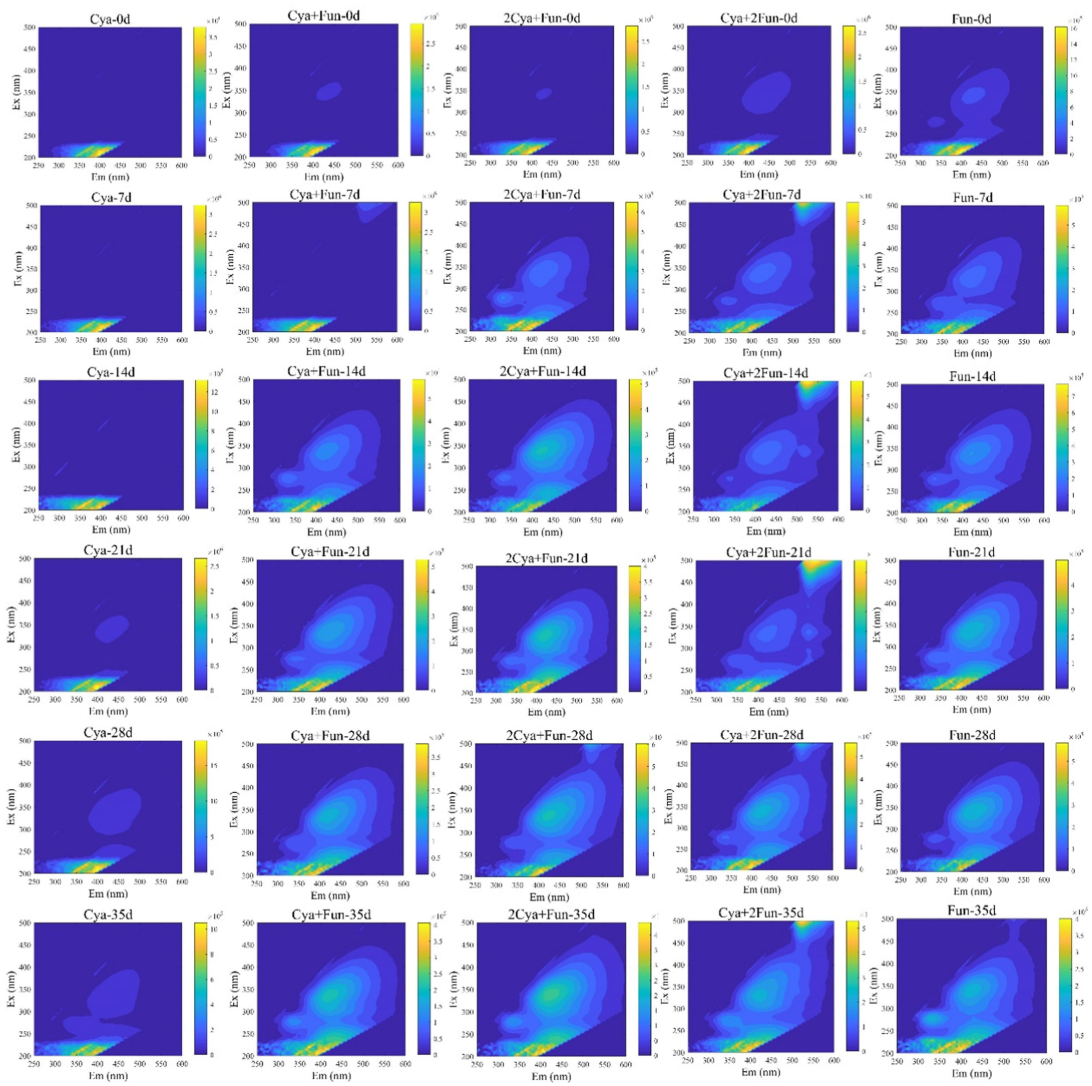
Dynamic changes in three-dimensional fluorescence spectra of DOM in cyanobacteria-fungi crusts with different inoculum biomass.

Dynamic variations in three-dimensional fluorescence spectra of DOM in cyanobacteria-fungi crusts with different inoculum biomass are depicted in Figure 2. The fluorescence intensity of each group exhibited a gradual increase during the cultivation process. In comparison with the Cya group, which was solely inoculated with *Phormidium tenue*, the soil DOM’s fluorescence intensity significantly increased after the addition of fungal Zh2, accompanied by the appearance of fluorescence peaks in the humus area (Em > 350 nm). The DOM fluorescence intensity in the Cya group was comparatively weaker in the humus, while cyanobacteria-fungi crusts displayed distinct fluorescence peaks in the humus area after 1 week of culture, gradually intensifying with the culture time. In the case of sole inoculation with Zh2, the fluorescence peak emerged in the humus area during the initial inoculation, and its intensity continued to increase in the later stages of the culture.

**Figure 3:**
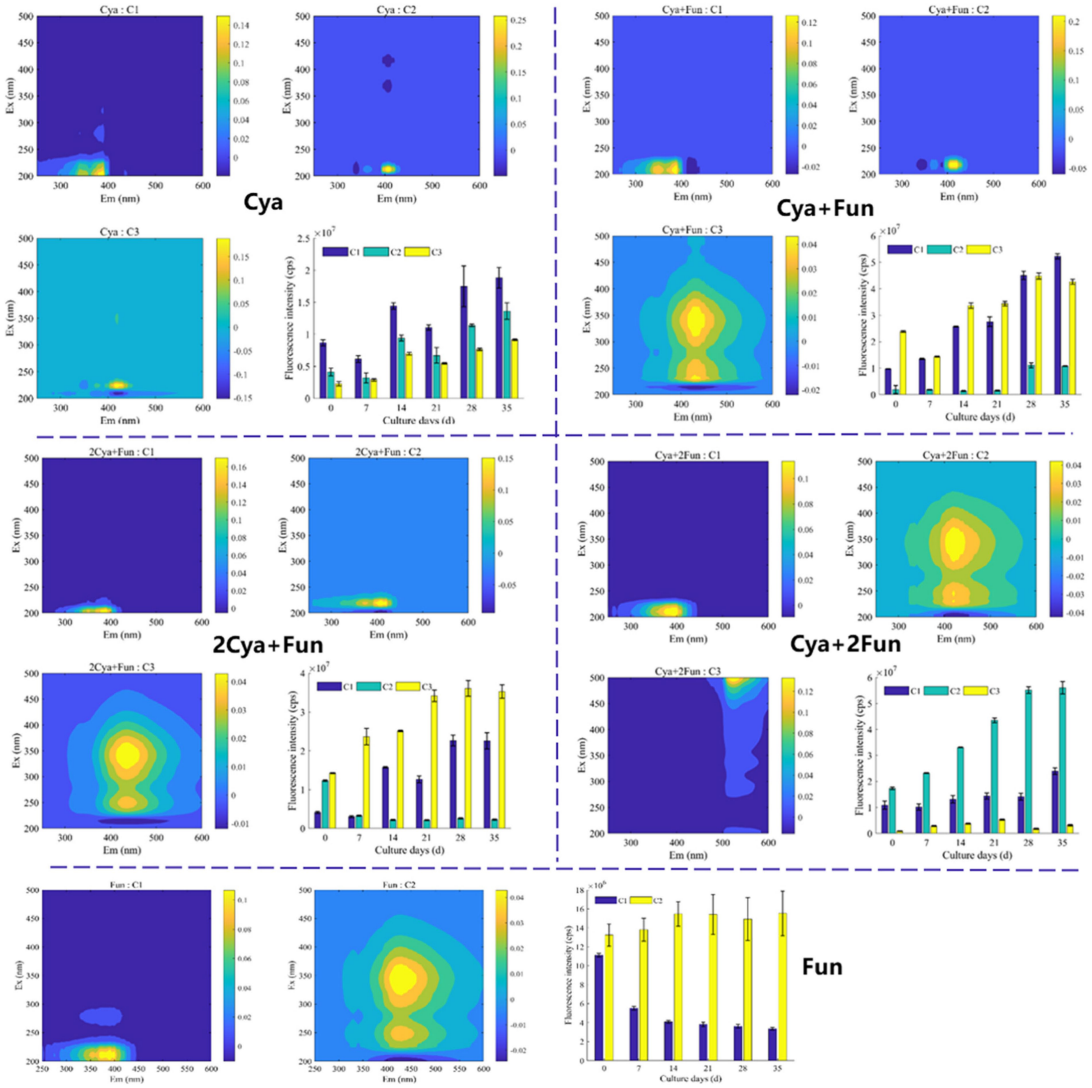
Spectral characteristics and fluorescence intensity of the two/three primary fluorescent components identified through EEM-PARAFAC analysis in cyanobacteria-fungi crusts with varying inoculum biomass.

For a more in-depth investigation into the photochemical characterization of DOM, PARAFAC analysis was performed to separate dissimilar fluorescent components from the DOM (Figure 3). Two fluorescent components were isolated from the DOM in the Fun group, and three components were obtained in the remaining four groups. The models for each component were consistent with previous reports or well-matched with the OpenFluor database, exhibiting similarity scores of >0.95. The C1 component (Ex/Em = 205–210/390–400) in the ultraviolet region, present in all five groups, was attributed to fulvic substances with low molecular weight. The C2 component (Ex/Em = 210–220/405–420) in the Cya, Cya + Fun, and 2Cya + Fun groups, and the C3 component in the Cya group (Ex/Em = 225/420), could be considered as fulvic-like substances. The C2 component in the Cya + Fun and 2Cya + Fun groups, and the C3 component in the Cya +2Fun and Fun groups (Ex/Em = 230–250, 340–345/420–435) belonged to the visible region and could be categorized as relatively stable humic-like substances with higher molecular weight. The fulvic-like component in DOM consisted of two components, with C1 present in all five treatment groups, showing small fluctuations in relative intensity. The C2 component only appeared in the Cya, Cya + Fun, and 2Cya + Fun groups. In comparison with the Cya group, the C2 component in the Cya + Fun and 2Cya + Fun groups exhibited a red-shift and decreased light intensity (Table 2).

**Table 2:** Position of DOM fluorescence peaks in cyanobacteria-fungi crusts with different inoculum biomass and a comparison with earlier reports from the OpenFluor database.

	C1		C2		C3	
	Wavelength (Ex/Em, nm)	Relative intensity	Wavelength (Ex/Em, nm)	Relative intensity	Wavelength (Ex/Em, nm)	Relative intensity
Cya	205/390	0.174	210/405	0.302	225/420	0.237
Cya + Fun	210/390	0.148	215/420	0.249	230,340/430	0.047,0.053
2Cya + Fun	205/390	0.195	220/410	0.186	250,340/435	0.039,0.051
Cya + 2Fun	210/400	0.131	345/420	0.054	500/525	0.154
Fun	210/390	0.123	250,345/430	0.039,0.052		
Component resemblances	Protein-like		Protein-like (except for Cya + 2Fun and Fun)		Humic-like (except for Cya)	
Open Fluor Study Matches	Borisover et al. (2011) and Derrien et al. (2019, 2020)				Lin and Guo (2020) and Zhuang et al. (2021)	

### 3.4 Soil DOC content and DOM fluorescence parameters

**Figure 4:**
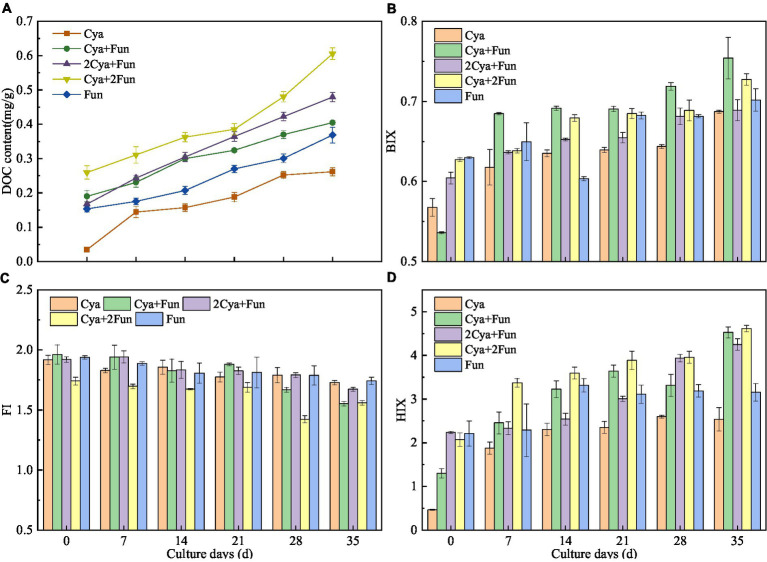
Dynamic changes in DOC content **(A)** and fluorescence parameters, including BIX **(B)**, FI **(C)** and HIX **(D)**, in cyanobacteria-fungi crusts with different inoculum biomass.

As shown in Figure 4, soil DOC content exhibited an upward trend with cultivation time in all crusts, but the accumulation rates varied with different cyanobacterial/fungal inoculation ratios. Following 35 days of inoculation, the soil DOC content demonstrated the following trend: Cya + 2Fun > 2Cya + Fun > Cya + Fun > Fun > Cya. Additionally, EEM was employed to assess the humification and bioavailability of DOM, as indicated by BIX, FI, and HIX. The BIX and HIX values of DOM exhibited significant differences in crusts with different inoculum biomass, while the FI values showed comparatively less variation. As the cultivation time increased, the BIX and HIX values of DOM demonstrated an upward trend, while the FI value decreased. After 35 days of cultivation, the BIX values of DOM in Cya + 2Fun and Cya + Fun were significantly higher than in the other groups. Moreover, the HIX values were notably higher in treatments involving the combination of cyanobacterial and fungal strains than in those of the Cya and Fun groups, which were inoculated solely with cyanobacteria or fungi.

### 3.5 Redundancy analysis between inoculum biomass and soil properties

**Figure 5:**
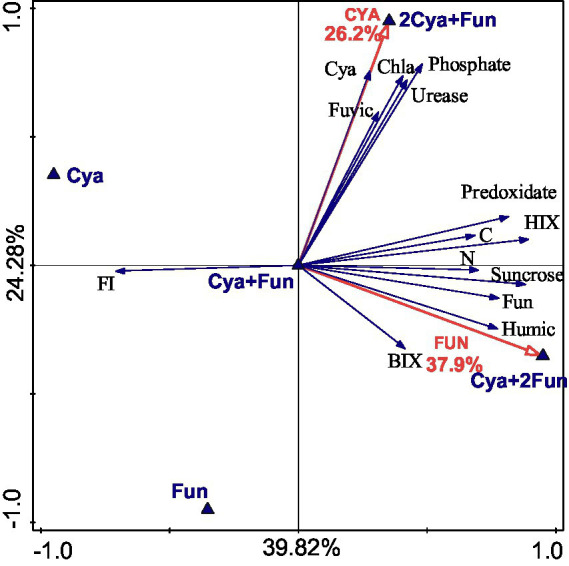
Redundancy analysis depicting the relationship between inoculum biomass and soil properties.

RDA was employed to explore the impact of inoculum biomass on crust growth and soil properties (Figure 5). The inoculation quantity of cyanobacteria accounted for 26.2% of the total variation in all crust and soil indices, while the fungal inoculum contributed to 37.9%. The five groups with different inoculum biomass exhibited an even distribution across four quadrants, with the Cya + Fun group situated in the middle and displaying correlations with the measured indicators. In addition to the expected positive correlation with Chl *a* content and cyanobacterial abundances, cyanobacterial inoculum demonstrated positive correlations with fulvic-like content, urease activity, and phosphatase activity. On the other hand, the quantity of fungal inoculum exhibited positive correlations with soil C, N content, catalase activity, invertase activity, and HIX, BIX value and humic-like content of soil DOM.

## 4 Discussion

The success of constructing the lichen bionic system relies on the potential mutual symbiosis between cyanobacteria and fungi (Frey-Klett et al., 2011; Scharnagl et al., 2023). The *Exophiala oligosperma* Zh2, belongs to Ascomycetes, which is consistent with the reported results that 90% of fungi in biological crusts are Ascomycetes (Bates et al., 2012; Abed et al., 2013). Fungi of the phylum Ascomycetes are found in the soil in parasitic, saprophytic, or symbiotic forms. In this study, *Exophiala oligosperma* Zh2 acting as a heterotrophic fungus, exhibited a gradual biomass decrease in the absence of external nutrient input (Figure 1). Co-inoculation with *Phormidium tenue* resulted in a continuous increase in fungal abundance in the soil, suggesting that *Phormidium tenue* could serve as a nutrient source for fungal growth. Concurrently, gene abundance and Chl *a* content of cyanobacteria increased with the addition of fungi, demonstrating a mutualistic symbiosis. Fungal Zh2 was characterized by high-yield exopolysaccharide, and exopolysaccharides are known to be beneficial for the growth of cyanobacteria and cyanobacterial BSCs (Colica et al., 2014; Rossi et al., 2018; Dabravolski and Isayenkov, 2022). Fungi transformed inorganic substances inaccessible to cyanobacteria into organic matter, while cyanobacteria supplied organic nutrients from photosynthesis to fungi (Guo and Tong, 2014; Li et al., 2022). The symbiotic dynamics in the cyanobacterial-fungal system were influenced by the inoculation ratio of cyanobacteria and fungi (Chu et al., 2021). After a 35-day culture, no significant differences were observed in cyanobacterial biomass between the 2Cya + Fun group and the Cya + Fun group, indicating that the inoculation ratio in the Cya + Fun group favored cyanobacterial-fungal symbiosis more than the 2Cya + Fun group. Cyanobacterial inoculation biomass positively influenced fungal growth to varying extents (Marks et al., 2019), with the promoting effect diminishing as the inoculum biomass increased. This could be attributed to an excess of cyanobacterial inoculum occupying the fungal niche, subsequently affecting fungal growth space (Zhang et al., 2014). An appropriate spatial structure is conducive to nutrient and gas exchange between microalgae and fungi, facilitating mutual growth (Li and Zhu, 2023).

Soil C, N, and enzyme activity serve as crucial indicators for restoring desert soil structure and function (Zhang et al., 2023). Significant differences in soil property indicators were observed between crusts with different cyanobacterial/fungal inoculation ratios (Table 1). In comparison to other groups, the C and N contents of the Cya + Fun group were higher, aligning with its elevated biomass, confirming the well-developed biological crust induced by the consortium of cyanobacteria and fungi. Soil enzyme activities, known for their sensitivity to environmental changes (Aponte et al., 2020; Wang et al., 2022), were notably higher after the coinoculation of cyanobacteria and fungi compared to single inoculation groups of Cya and Fun, showcasing the contribution of the cyanobacteria-fungi crust to soil enzyme activities. Furthermore, the Cya + Fun group exhibited the highest activities of catalase and sucrase. Catalase, an oxidoreductase in soil, is frequently employed to characterize soil humification intensity and organic matter conversion rate. It plays a pivotal role in the oxidation of organic matter and humus formation (Liu et al., 2020). The significantly higher catalase activity in the Cya + Fun group reflected its robust microbial activity (Prasanna et al., 2012). Soil sucrase enhances soluble nutrients in soil and is closely associated with the transformation of organic matter and respiration intensity (Xiao et al., 2022; Yang et al., 2024). In this study, sucrase activity notably increased with fungal addition, possibly linked to the substantial exopolysaccharide secretion by *Exophiala oligosperma* Zh2. Consequently, the cyanobacterial-fungal mixture in this study may contribute to humus formation and the transformation of organic matter structure.

**Figure 6:**
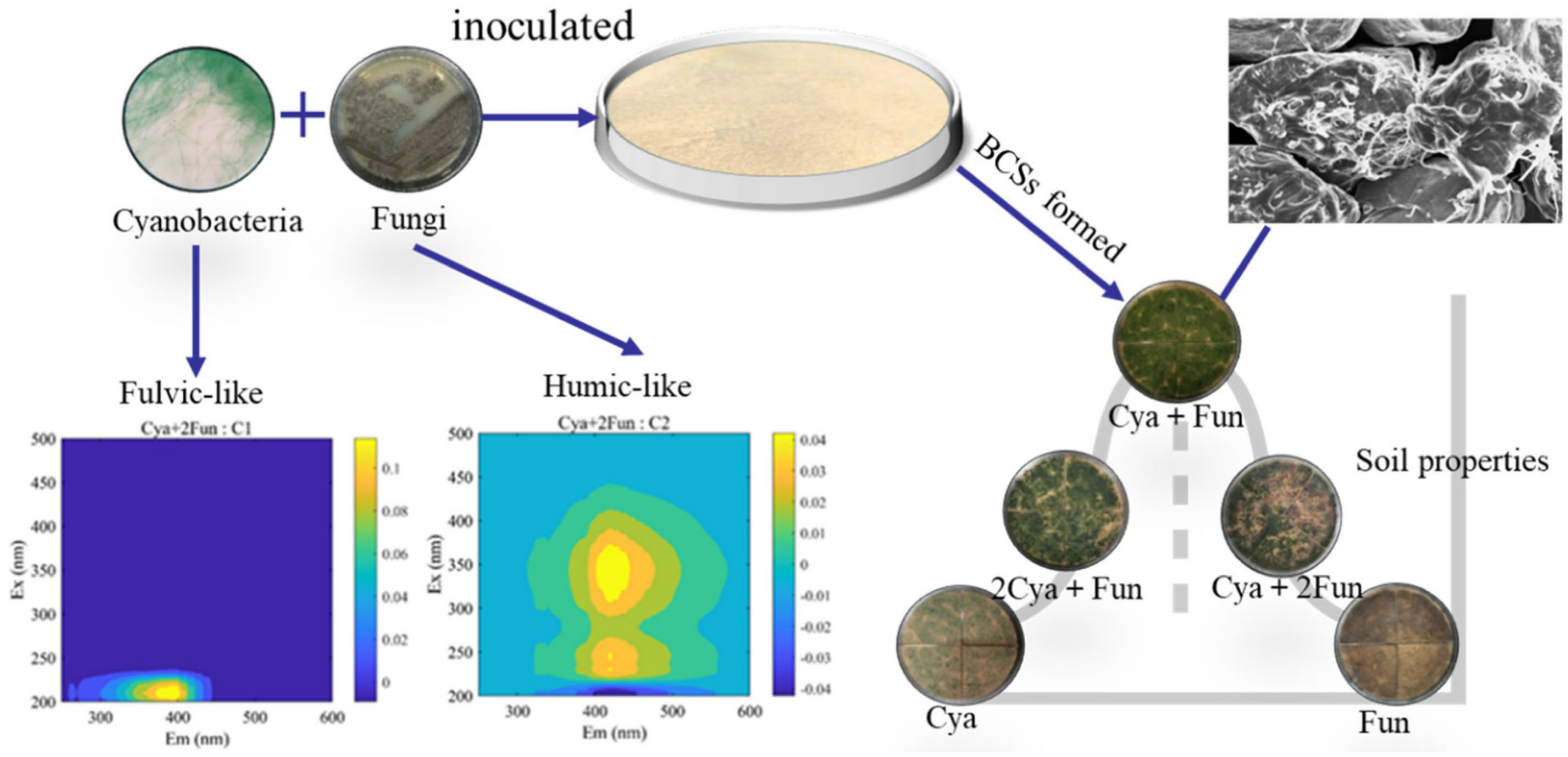
The collaborative impact of cyanobacteria and fungi enhances the accumulation of dissolved soil organic matter in the crust.

Soil humus, derived mainly from microbial metabolism and biotransformation, plays a pivotal role in regulating soil carbon and nitrogen cycling, microbial growth, and structural stability in ecological processes (Schmidt et al., 2011; Vikram et al., 2022). It serves as a crucial indicator for assessing the effectiveness of degraded soil restoration. Throughout the incubation process, the fluorescence intensity of DOM in all groups increased, indicating that DOM mainly originated from microorganisms such as cyanobacteria and fungi in the soil (Meng et al., 2016; Li et al., 2023). Despite a decreasing trend in fungal biomass in the later stages of cultivation (Figure 1), the fluorescence intensity of soil DOM continued to rise (Figure 2). This could be attributed to the continuous accumulation of fungal metabolites during the cultivation process, with the decrease in fungal biomass having a relatively minor impact on the metabolic capacity of the remaining viable fungi. Two components—fulvic-like and humic-like—were isolated from the artificial crust DOM. Fulvic-like substances, characterized by hydrophilic small molecules, dispersed uniformly in solution at any pH, displaying strong diffusivity and instability (Canellas et al., 2015). In contrast, humic-like substances, rich in aromatic groups, indicated higher maturity and humification, forming more stable bonds with the mineral components of the soil (Mohinuzzaman et al., 2020). The high-molecular-weight humic-like component (Ex/Em = 230, 340/430 nm) was exclusively present in treatment groups with fungal inoculation, emphasizing the fungus Zh2’s capacity to contribute humic substances to soil DOM through its metabolic activities (Figure 6). The humic-like component C3 appeared in cyanobacterial-fungal and fungal crusts, albeit with relatively low intensity, possibly due to the high molecular weight and weak fluorescence intensity of humic-like substances. Besides humic components, two fulvic components were identified. Fulvic component C1 (Ex/Em = 210/390 nm) appeared in all five treatment groups with minor intensity fluctuations, suggesting it might represent a small amount of low-molecular-weight organic compounds in the sand matrix. Fulvic component C2 (Ex/Em = 210/405 nm) exhibited higher fluorescence intensity in the Cya group than in the Cya + Fun and 2Cya + Fun groups after fungal addition, indicating its potential origin from the metabolic products of filamentous cyanobacteria. Fulvic components displayed a redshift trend after fungal inoculation, typically associated with increased molecular conjugation effects and enhanced molecular condensation (Yakimov et al., 2021). This suggested an increase in the aromaticity and complexity of fulvic component C2, leading to higher humification and structural complexity post-fungal inoculation. Given that the cyanobacterial-fungal crust exhibited higher levels of humic-like and relatively lower levels of fulvic compared to the single algal crust, fungal inoculation may facilitate the gradual transformation of fulvic into humic, enhancing soil humification and stability.

Soil DOC primarily originated from microbial metabolic activities and constituted an important component of total soil carbon, thus being significantly correlated with soil organic carbon content (Zhang et al., 2018). However, this study revealed inconsistent patterns between soil C and DOC content. The highest DOC content was observed in the Cya + 2Fun and 2Cya + Fun groups, while the highest C content was found in the Cya + Fun group. This discrepancy suggested that the Cya + Fun group might contain a higher proportion of water-insoluble organic compounds, such as humin. Humic substances, particularly humin, represent the most tightly bound fraction of organic matter to mineral components. Under certain conditions, like dryness, humic acid can transform into humin irreversibly (Schmidt et al., 2011). Humin constitutes the majority of soil organic carbon, playing a crucial role in soil fertility and the ecological environment (Pham et al., 2021). However, further research is needed to confirm whether the Cya + Fun group indeed contains a higher proportion of humin components. Changes in fluorescence parameters can reflect the characteristics of DOM. This study found that parameters BIX and HIX were more sensitive in reflecting DOM characteristics compared to FI, consistent with other studies (Zhang et al., 2018). Throughout the incubation process, the bioavailability and humification degree of crust DOM exhibited an increasing trend, confirming the contribution of cyanobacteria and fungi to soil humus. Research has demonstrated that the artificial addition of humic and fulvic acids to the soil can effectively enhance nutrient bioavailability (Delgado et al., 2002). The enhancement of humic and fulvic through cyanobacteria and fungal inoculation played an irreplaceable role in improving soil fertility, activity, and stability.

The effects of algal and fungal inoculation on composite crust biomass and soil properties were examined based on the RDA analysis. Both cyanobacteria and fungi significantly contributed to crust biomass and soil properties, but their main targets of action were different. Inoculation with cyanobacteria had a pronounced promoting effect on the content of fulvic, and the activities of urease and phosphatase. Research on the detection of humic acids released during the growth cycle of *Microcystis* found only fulvic components and did not detect the presence of humin components (Yilu et al., 2015), which was consistent with the significant correlation between cyanobacterial inoculation and fulvic content observed in this study. Moreover, the inoculation of the high exopolysaccharide-producing fungus Zh2 showed a significant positive correlation with humic-like component content. Furthermore, fungal inoculation, rather than cyanobacterial, significantly correlated with soil carbon content. This could be attributed to the fact that most of the carbon fixed by filamentous cyanobacteria through photosynthesis was stored in the soil by filamentous fungi, which could convert a substantial amount of new carbon into stable carbon (Schmidt et al., 2011). This also explained the positive correlations observed between measured crust biomass and soil properties in the Cya + Fun group. In the 2Cya + Fun group, with a higher abundance of cyanobacteria, the soil humus was predominantly composed of fulvic, indicating insufficient humification degree and stability. In the Cya + 2Fun group, with a higher abundance of fungi, the ecological niche of filamentous cyanobacteria was occupied. Filamentous cyanobacteria, as photosynthetic organisms, could only grow in the surface layer of the soil. Kheirfam and Roohi (2020) inoculated bacteria and cyanobacteria on the dried-up lakebeds to induce the formation of biological soil crusts. Their results revealed that the inoculation of the bacteria, separately or in combination with cyanobacteria, had a limited effect on accelerating crust formation. The co-inoculation of cyanobacteria and fungi in this study exhibited a synergistic effect, possibly due to the mutualistic symbiosis between cyanobacteria and fungi. The reduced photosynthetic efficiency of cyanobacteria directly affected the input of new carbon into the soil, indirectly influencing the abundance of fungi and the synthesis of stable carbon in the soil. Therefore, the cyanobacterial-fungal inoculation in the Cya + Fun group was considered to achieve the optimal synergistic effect in the symbiotic system. Chaudhary et al. (2020) co-inoculated biological crust fragments and arbuscular mycorrhizal (AM) fungi into the arid land simultaneously, but unexpectedly, no synergistic effect emerged. The authors attributed this to the presence of AM fungal propagules in the background soil and inoculated biological fragments, eliminating the need for additional AM fungal inoculation. However, in this study, the abundance of sand dune fungi was low, and we inoculated cyanobacterial inoculants, necessitating some fungal supplementation to help construct a richer soil species ecological structure.

## 5 Conclusion

The study explored the impacts of varying inoculation biomass on the formation and development of cyanobacterial-fungal composite crusts. The findings revealed noteworthy differences in growth conditions and soil nutrient content across different inoculation scenarios. Cyanobacteria inoculation substantially increased the content of the fulvic-like component in the soil, whereas fungal inoculation markedly elevated soil humic-like content and humification degree. Cyanobacterial inoculation demonstrated a positive correlation with fulvic-like content, urease, and phosphatase activities in the soil. Fungal inoculation exhibited a significantly positive correlation with soil C and soil N content, catalase and sucrase activities, as well as HIX and BIX. Hence, cyanobacteria and fungi played distinct roles in the cyanobacterial-fungal symbiotic system, contributing differentially to soil nutrition and stability. The optimal synergistic effect of the cyanobacterial-fungal symbiotic system was observed with an appropriate initial proportion of cyanobacterial and fungal inoculation, as evidenced by the Cya + Fun group. This study furnishes crucial theoretical support for the co-inoculation of cyanobacteria and fungi in desertification treatment.

## Data availability statement

The datasets presented in this study can be found in online repositories. The names of the repository/repositories and accession number(s) can be found at: https://www.ncbi.nlm.nih.gov/genbank/, accession number: MH973233.

## Author contributions

XZ: Conceptualization, Data curation, Formal analysis, Funding acquisition, Methodology, Visualization, Writing – original draft, Writing – review & editing. BL: Data curation, Formal analysis, Methodology, Writing – original draft, Writing – review & editing. TZ: Data curation, Methodology, Writing – original draft, Writing – review & editing. QX: Formal analysis, Writing – original draft, Writing – review & editing. XM: Methodology, Writing – original draft, Writing – review & editing. LC: Conceptualization, Funding acquisition, Project administration, Resources, Writing – original draft, Writing – review & editing.

## Footnotes

^1^ http://www.models.life.ku.dk/

^2^ https://openfluor.lablicate.com
