# Supplementary material for: Co-inoculation of fungi and desert cyanobacteria facilitates biological soil crust formation and soil fertility
Source: Front Microbiol. 2024 Apr 8;15:1377732. doi: 10.3389/fmicb.2024.1377732 (PMC11033444; doi:10.3389/fmicb.2024.1377732)
Supplement: Supplementary file 1 [file Data_Sheet_1.pdf]

Supplementary Material

Table S1. The main characteristics of the studied sand

Clay (%)	Silt (%)	Sand (%)	Bulk density (g cm^{-3})	pH	EC (μs cm^{-1})
0.37±0.02	27.9±0.75	71.36±1.47	1.645±0.132	7.91±0.15	72.6±2.4

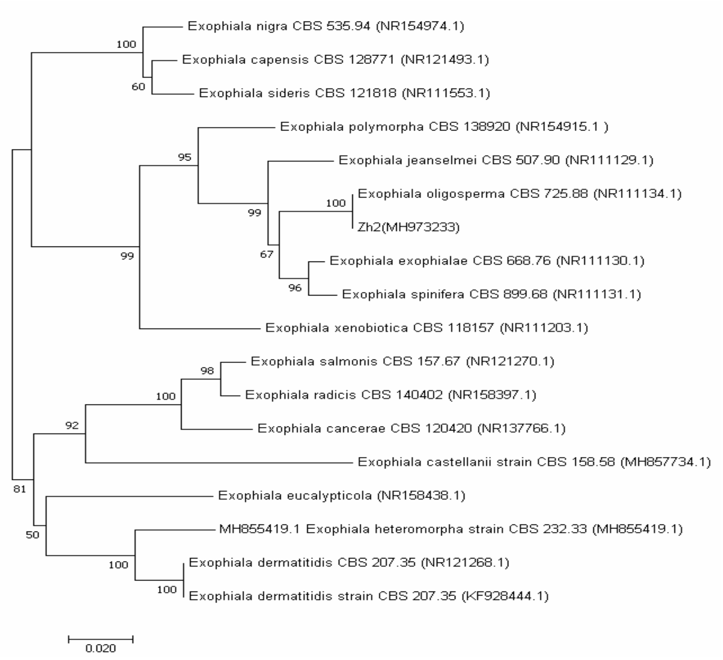

Fig.S1. Phylogenetic dendrogram of *Exophiala oligosperma* Zh2 obtained by the ITS sequences.
